# Polyfluoroalkyl-Substances Detection in Junk Food Packing Materials Using Various Analytical Methods: A Review

**DOI:** 10.7759/cureus.70301

**Published:** 2024-09-27

**Authors:** Jayanthy S, Kanaka Parvathi Kannaiah, Damodharan Narayanasamy

**Affiliations:** 1 Pharmaceutical Quality Assurance, SRM College of Pharmacy, SRM Institute of Science and Technology, Chennai, IND; 2 Pharmacy, SRM College of Pharmacy, SRM Institute of Science and Technology, Chennai, IND; 3 Pharmaceutics, SRM College of Pharmacy, SRM Institute of Science and Technology, Chennai, IND

**Keywords:** analytical approaches, extraction techniques, food packaging materials, junk food, per- and polyfluoroalkyl substances

## Abstract

Per- and polyfluoroalkyl substances (PFAS) are unseen, dangerous organic compounds that can cause major health disorders. PFASs have been categorized as persistent, bioaccumulative, and toxic (PBT). This review provides knowledge about the PFASs present in junk food packaging materials, which can migrate into the food. Different types of samples were analyzed using the analytical methods. The most preferred method of extraction is ultrasonic-assisted extraction (UAE). It summarizes the analytical approaches of PFASs. The results of numerous studies show that perfluorooctanoic acid is the most often detected compound with high concentrations. The European Food Safety Authority (EFSA) announced that the tolerable weekly intake (TWI) of PFASs is 4.4 ng/kg. The US Environmental Protection Agency (EPA) has announced the limit for perfluorobutane sulfonic acid due to its toxicity level. These compounds have potential effects on both people’s health and the biosphere. PFAS usage has to stop in the industries for a better future.

## Introduction and background

**Figure 1:**
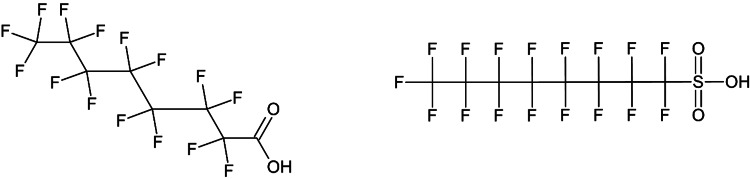
Structure of perfluorooctanoic acid and perfluorooctane sulfonic acid Image Credit: Created by the authors using Chemsketch

Per- and polyfluoroalkyl substances (PFAS) are synthetic organic chemicals (Figure 1) [1] and belong to the perfluorocarbons (PFCs) family [2]. Discovered in 1930, these substances were synthesized using two methods: electrochemical fluorination and fluorotelomerization. Since the 1950s, PFAS have been widely used in various industrial applications, including firefighting equipment, consumer products, cosmetics, paints, furniture, and food packaging materials (FPM), due to their resistant properties [3]. They are resistant to oil, water, stains, grease, and heat and are both hydrophobic and lipophobic [4]. The PFAS family includes more than 4,700 individual compounds [5]. These chemicals are highly persistent in the environment and are difficult to break down due to the strong carbon-fluorine bonds in their structure, earning them the label “forever chemicals” [6,7]. PFAS have significant environmental and health impacts, causing issues such as genotoxicity, cardiovascular diseases, cancer, immunological disorders, and reproductive problems [8]. In humans, the approximate serum half-lives of perfluorooctane sulfonic acid (PFOS), perfluorohexane sulfonate (PFHxS), and perfluorooctanoic acid (PFOA) are 3.5 years, 4-5 years, and 5.3 years, respectively [9].

PFAS can migrate into food from FPM, particularly at high temperatures. Typically, 100 ppm of these substances are added to packaging materials. Fast food products have been found to contain the highest levels of perfluorooctane sulfonamide, at 27.3 µg/kg. Additionally, microwave popcorn bags contain 3-4 mg/kg of PFAS, with 300 µg/kg detected in the popcorn itself. The European Union (EU) has set a regulatory limit for PFOS and its salts at 0.025 mg/kg [2]. In the United States, approximately 940 tons of PFAS have been reported in FPM. Due to their harmful effects, some of these substances have been banned [10].

Since 2009, PFOS and its salts have been listed in Annex B of the Stockholm Convention because of their persistence and harmful effects, including perfluorooctane sulfonyl fluoride [11]. The EU's REACH regulation (Registration, Evaluation, Authorization, and Restriction of Chemicals) also restricts the use, manufacture, import, and export of certain PFAS [3]. PFOS and its salts are specifically banned under EU regulations. According to the US Food and Drug Administration, wastewater from typical paper mills exposed around 40 to 100 kg of PFAS per day in 2018 [12].

In 2020, Denmark prohibited the use of cardboard and paper containing PFAS in food packaging [5]. The European Chemicals Agency (ECHA) classified polyfluoroalkyl compounds and their salts as substances of very high concern in 2020 [13]. The Danish Ministry of Environment and Food also banned the use of fluorinated materials in paper and board food packaging without an effective barrier to prevent migration into food [7].

In 2021, after evaluating the toxicity of perfluorobutane sulfonate, the US Environmental Protection Agency (EPA) set limits for chronic and sub-chronic exposure at 0.0003 and 0.001 mg/kg body weight/day, respectively [13]. By 2022, 11 US states had passed laws prohibiting the addition of PFAS to food packaging [5]. In 2023, five EU Member States submitted a proposal to the ECHA to ban the manufacture, distribution, and use of PFAS [7].

The Canadian Environmental Protection Act restricts the production, use, sale, or import of perfluorooctanoic acid (PFOA), long-chain perfluoroalkyl carboxylic acids (PFCAs), and their salts [11]. According to the European Food Safety Authority (EFSA), the combined exposure limit for four specific PFAS (PFOA, perfluorononanoic acid, PFOS, and perfluorohexane sulfonate) should not exceed 4.4 ng/kg body weight per week, with packaging materials contributing 17.5 to 45.9 µg/kg [7,9]. EFSA has also established tolerable daily intakes (TDI) of 150 ng/kg for PFOS and 1,500 ng/kg for PFOA, declaring PFAS as emerging contaminants in the food chain [11].

This review aims to identify per- and polyfluoroalkyl compounds, explore their applications, and examine their effects. Various analytical techniques were employed to determine the concentrations of these substances, and the results are discussed in detail.

## Review

Extraction of PFAS in FPM

**Figure 2:**
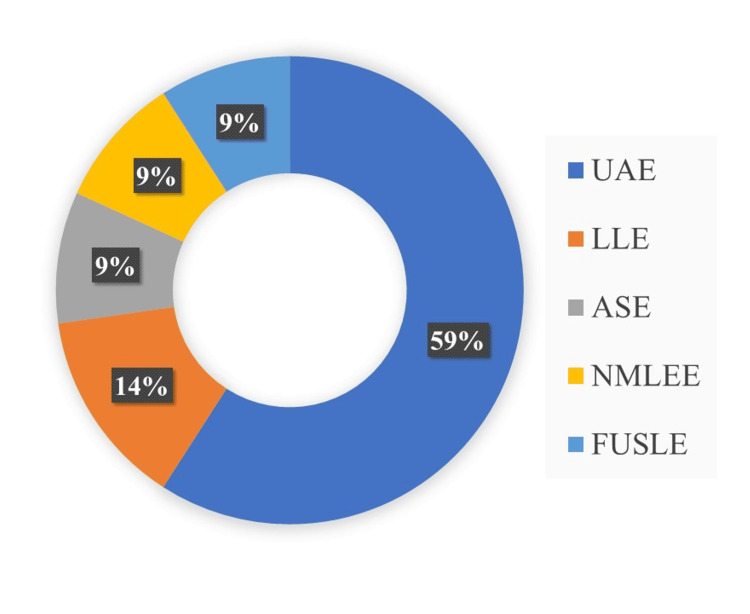
Different extraction methods used for the determination of per- and polyfluoroalkyl substances Image Credit: Original illustration. Created with MS Word UAE: ultrasonic-assisted extraction; LLE: liquid-liquid extraction; ASE: accelerated solvent extraction; NMLEE: non-microwave liquid-liquid extraction; FUSLE: focused ultrasound-assisted solvent extraction

The process of isolating active or selective compounds from a solid using liquid solvents is a crucial technique for separating various chemicals in a mixture. Figure 2 presents an overview of the different extraction methods used to determine PFAS. These compounds were analyzed and detected using a range of extraction techniques.

Extraction using ultrasonic-assisted extraction (UAE)

Ultrasound-assisted extraction (UAE) is a method that uses ultrasonic waves to extract PFAS with reduced energy and solvent consumption. Compared to other extraction procedures, this technique is known for its cost-effectiveness and ease of use.

Chen et al. developed an extraction method in which paper samples were collected and cut into uniform pieces, and 0.5 g of each sample was weighed, with the remainder stored in polyethylene bags. The weighed sample was transferred to 15 mL polypropylene tubes containing 10 mL MeOH and internal standards. After vortexing for two minutes at 2,500 rpm, the tubes were ultrasonicated at 40°C for one hour, followed by centrifugation for 10 minutes at 3,000 rpm. A 5 mL aliquot of the supernatant was transferred to a sterile polypropylene tube, concentrated using nitrogen gas, and reconstituted in MeOH. Prior to analysis, the extract was filtered through polypropylene microfilters and analyzed using UHPLC-MS [10].

Vera et al. applied the UAE method to samples of cardboard used for packaging pizza, sandwiches, bakery paper, and recycled cardboard for hot dogs. Five successive extractions were performed using 3 mL ethanol over one hour, combined, and dried under nitrogen gas. The extracts were analyzed using UHPLC-IMS-QTOF [14].

Langberg et al. conducted a study using 0.1 g samples of small pieces of recycled paper, mixed with ethyl acetate, ultrasonicated at 35°C for 45 minutes, and centrifuged for 10 minutes. The procedure was repeated twice for additional extracts. The final extract was purified with Milli-Q water, centrifuged again, dried under nitrogen gas, and reconstituted with a 50:50 MeOH solution. The extracts were analyzed using UHPLC-QqQ-MS [12].

Hoang et al. used UAE extraction on samples such as instant noodle cups, mochi paper trays, cookie wrappers, chip wrappers, paper cups, potato snack boxes, and hamburger wrappers. Printed layers were removed and the materials were cut into small pieces. A 1 g sample was placed in a polypropylene centrifuge tube with MeOH and ultrasonicated for two hours at 60°C. After centrifugation, the supernatant was transferred to a glass round-bottom flask, and the extraction process was repeated. The combined extracts were evaporated using a rotary evaporator, dried under nitrogen gas, reconstituted in MeOH, filtered using a nylon membrane, and analyzed by LC-MS [1].

Stroski et al. used UAE on plastic food storage bags as samples. The samples were placed in polypropylene tubes with MeOH and shaken for one hour using a Glas-Col shaker. After sonication for one hour, the extract was centrifuged, transferred to a polypropylene tube, dried under nitrogen gas, reconstituted with MeOH, sonicated for five minutes, and centrifuged again for three minutes. The extracts were transferred to polypropylene vials and analyzed using LC-HRMS [13].

Dueñas-Mas et al. optimized an extraction method for microwave popcorn bags, cardboard cups, baking paper, burger wrappers, muffin cups, ice cream cups, and takeaway paper bags. Samples were chopped into fragments, and 2 g of each was placed in a polypropylene tube with internal standards. Extraction was performed twice (30 minutes each) at room temperature using sonication with MeOH solvent. The combined extracts were dried under nitrogen gas and diluted with 1.5 mL of a MeOH solution containing 0.3% acetic acid. The extracts were purified using a pre-conditioned ENVI-Carb cartridge, eluted, dried, reconstituted with MeOH, diluted with water, and transferred to chromatographic vials for further analysis using LC-MS [3].

Although Chen et al., Stroski et al., Langberg et al., Hoang et al., and Dueñas-Mas et al. all used the UAE extraction method, Vera et al. employed five successive extractions before combining and analyzing the samples using LC-MS/UHPLC-MS. These extraction methods enable accurate analysis of PFAS.

Advantages

Reduces the time needed for PFAS extraction compared to traditional methods. It requires less solvent than conventional methods, reducing environmental impact, lowering costs, and minimizing solvent handling hazards. The combination of UAE with green solvents or reduced solvent volumes aligns with sustainable practices and environmentally friendly approaches.

Limitations

Less effective for short-chain compounds, which are more hydrophilic and less likely to interact well with organic solvents during the extraction process. Ultrasonic equipment may degrade over time due to the high energy it generates, leading to maintenance issues or reduced performance.

Extraction using focused ultrasonic solid-liquid extraction (FUSLE)

A novel method for removing chemicals from solid matrices uses ultrasonic energy and serves as an alternative to UAE, employing an ultrasonic probe instead of a water bath.

Zabaleta et al. optimized this method using microwave popcorn bags as samples. The samples were cut into uniform pieces and placed in a vessel containing MeOH. Extraction was carried out in an ice-water bath, followed by filtration using polyamide filters, and the extract was reduced to 0.5 mL under nitrogen gas. The purified extract was processed through a pre-conditioned ENVI-Carb cartridge. After elution, the extract was dried under nitrogen gas, reconstituted in MeOH, and filtered again using polypropylene microfilters before being analyzed by LC-QToF [11].

Advantages

It promotes the breakdown of solid matrices, which can improve the extraction of PFAS compared to conventional techniques. This is especially effective for tightly bound PFAS. It can be applied to a wide range of sample sizes from small lab samples to larger-scale extractions.

Limitations

It can be expensive and may not be readily available in all laboratories, making it less accessible than simpler like liquid-liquid extraction (LLE). High-intensity ultrasonic energy can lead to some analytes or co-extracted compounds, although PFAS are generally stable. Careful optimization of ultrasonic intensity and duration is needed to avoid unwanted effects.

Extraction using LLE

Surma et al. employed a method to extract PFASs from various samples, including wrapping papers, roasting bags (brands A, B, and C), and baking papers. The samples were chopped into small fragments and placed in a vial containing 80 mL of acetonitrile. The mixture was shaken at 250 rpm for one hour. The extracts were collected, evaporated to dryness using a rotary evaporator, and then reconstituted with 1 mL of MeOH. After a 15-minute centrifugation, the extract was diluted five times with acidified deionized water before infusion and analysis by LC-MS [2].

Bugsel et al. optimized a similar extraction method for PFAS. Paper samples were cut into rectangular pieces, placed in a beaker with 100 mL of MeOH, covered with transparent foil, and left overnight. The extracts were then dried at 40°C using a rotary evaporator, transferred to glass vials, concentrated under nitrogen gas, and stored in polypropylene vials for further analysis by LC-HRMS [4].

While both Surma et al. and Bugsel et al. used similar extraction methods, Surma et al. immediately diluted the extract five times and analyzed it, whereas Bugsel et al. soaked the samples in MeOH overnight before extraction and analysis.

Advantages

It is efficient at extracting PFAS, particularly long-chain compounds, from water due to their hydrophobic and oleophobic nature, which makes them partition into organic solvents. It can be applied to various types of samples, including water, soil, and biological samples, making it a versatile method for PFAS analysis.

Limitations

It can be less efficient for short-chain PFAS, which are more hydrophilic and have lower partition coefficients in organic solvents. It requires the use of organic solvents, which can be toxic and hazardous, raising concerns about waste disposal and environmental impact.

Accelerated solvent extraction (ASE)

Jovanović et al. used microwave popcorn bags, pizza boxes, muffin cups, sandwich wrappers, and coated paper as samples. These materials were cut into uniformly sized pieces. A 2 g portion of each sample was placed in ASE cells, securely sealed, and subjected to MeOH extraction cycles at 90°C. The resulting extracts were collected in evaporation vials, dried under nitrogen gas using the Biotage TurboVap II evaporation system, and then reconstituted with 5 mL of MeOH. The extracts were filtered through syringe filters into polypropylene autosampler vials and analyzed using LC-MS [7].

Advantages

It reduces the time required for extraction compared to traditional methods, often taking 15-30 minutes per sample. Less solvent is required due to the high efficiency and making it more environmentally friendly.

Limitations

The challenges may be some complex matrices may interfere with extraction efficiency, so proper method optimization is required and contamination can occur from various sources during sampling, handling, and analysis, so strict quality control measures are necessary.

Nonaqueous miscible liquid-liquid electroextraction (NMLEE)

NMLEE is an effective method for analyzing multiple PFAS using electric field-assisted extraction without the need for internal standards (IS).

Yuan et al. applied this extraction method to samples including instant noodle cups, plastic bags, popcorn bags, baking paper, fried food packaging, and tinfoil. The samples were cut into pieces and the required amount was diluted with a mixture of methyl tert-butyl ether (MTBE), methanol (MeOH), and acetonitrile. After shaking well, the cathode and a PEEK tube were immersed in the sample. Electroextraction was then performed, and both the cathode and PEEK tube were removed. The resulting samples were analyzed using LC-MS [15].

Advantages

The combination of an electric field with miscible solvents allows for highly selective extraction of PFAS, potentially avoiding issues with matrix interferences commonly encountered in traditional LLE. It uses smaller amounts of solvent, making it more environmentally friendly and reducing the cost of solvent disposal.

Limitations

Solvent compatibility is necessary since two miscible solvents must still allow for selective partitioning under electroextraction conditions. PFAS compounds vary in chain length and functional groups, so method optimization is needed to ensure efficient extraction for both short-chain and long-chain PFAS.

Identification and determination of PFAS

Many analytical methods are available now, yet liquid chromatography connected to a mass spectrometer and its associated methods are widely used to estimate the PFAS.

High-performance liquid chromatography with mass spectrometer

Bugsel et al. used a 1290 HPLC system connected to a 6550 QTOF mass spectrometer to analyze and detect PFAS. Paper samples were extracted, and the resulting solution was analyzed using a C18 column with a particle size of 2.7 µm. The process was conducted at 40°C with a flow rate of 0.4 mL/min. Gradient elution was employed with a mobile phase consisting of H2O/MeOH with NH4Ac (phase A) and MeOH/H2O with NH4Ac (phase B), both in a 95:5 v/v ratio, with the concentration increasing linearly from 25% to 85%. The sample was injected at 20 µL and analyzed in negative ionization mode over a scan range of m/z 100-m/z 1700. Major contaminants identified were perfluoroalkyl carboxylic acids (PFCAs) with carbon chain lengths C7 and C8, and fluorotelomer sulfonic acids (FTSAs) with chain lengths C6 and C8. A total of 14 PFAS from eight different compound classes were analyzed and detected [4].

Vavrouš et al. employed an HPLC-MS method to analyze PFAS in extracts from paper bags and cardboard samples. The HPLC system was coupled to a triple quadrupole mass spectrometer, using a reverse-phase C18 column with a particle size of 2.7 µm. The mobile phase included phase A, ammonium acetate in ultrapure water, and phase B, ammonium acetate in MeOH. The sample was injected at 20 µL with a flow rate of 0.4 mL/min. Recovery rates ranged from 70% to 120% [16].

Simonetti et al. utilized the HPLC-ESI-Q-TRAP method to analyze samples of microwave trays and baking papers, which were extracted prior to analysis. The isolation process used reverse-phase methodology with a mobile phase consisting of eluent A (ammonium acetate) and eluent B (MeOH, 45:55 ratio) at a flow rate of 0.2 mL/min. The analysis was conducted in MRM mode at 35°C for 20 minutes with a 5 µL injection volume. The majority of detected PFAS were short-chain compounds (C4 - C6), with perfluorooctanoic acid identified in concentrations ranging from 0.004 to 0.099 mg/kg [17].

Liquid chromatography with high-resolution mass spectrometer (LC-MS/HRMS)

Table 1 depicts the analytes, analytical conditions, and their results. Surma et al. used LC-HRMS to analyze PFAS in samples of wrapping papers and baking papers. The samples were extracted, evaporated, and centrifuged before analysis. The LC-HRMS system, equipped with a triple quadrupole, an ESI ion source, and an ion trap, operated at 45°C with an XBridge C18 column. Gradient elution was performed with solvent A (water/formic acid) and solvent B (acetonitrile/formic acid), both at a 99.0/1.0 ratio, with a flow rate of 0.23 mL/min in MRM mode. Data were processed using ANALYST 1.5.1 software. The analysis detected 10 PFCs, including seven perfluoroalkyl carboxylates (PFCAs) and three other PFAS. Perfluorooctanoic acid was the most frequently detected compound, with concentrations ranging from 0.10 to 6.22 pg/cm^2^ [2].

**Table 1:** Analytes with analytical conditions and their result HPLC-QTOF-MS: high-performance liquid chromatography-quadrupole time-of-flight tandem mass spectrometry; LC-MS: liquid chromatography with mass spectrometer; LC-HRMS: liquid chromatography with high-resolution mass spectrometer; HPLC-ESI-Q-TRAP: high-performance liquid chromatography with electron spray ionization-triple-quadrupole-linear ion trap mass spectrometer; UHPLC-MS-QTOF: ultra-high performance liquid chromatography-quadrupole time-of-flight tandem mass spectrometry; PFCAs: perfluoroalkyl carboxylic acids; FTCAs: fluorotelomer carboxylic acids; FTUCAs: fluorotelomer unsaturated carboxylic acids; PFHxA: perfluorohexanoic acid; PFTrDA: perfluorotridecanoic acid; PFTeDa: perfluorotetradecanoic acid; PFUdA: perfluoroundecanoic acid; diPAP: disubstituted polyfluorinated alkyl phosphate ester; FTSAs: fluorotelomer sulfonic acids; PFPeA: perfluoropentanoic acid; PFHpA: perfluoroheptanoic acid; PFDoS: perfluorododecane sulfonate; PFSAs: perfluorosulfonic acids; PFODA: perfluorooctadecanoic acid; PFDoDA: perfluorododecanoic acid; PFHxDA: perfluorohexadecanoic acid; PFDS: perfluorodecanoic sulfonate; PFS: perfluoro sulfonates

Detection technique	Conditions	Target analytes	No. of analytes detected	Results	Reference
HPLC-QTOF-MS	C18 column Particle size - 1.8 µm Temp - 40˚C	57 per- and polyfluoroalkyl substances	26 per- and polyfluoroalkyl substances (13 PFCAs, 5 FTCAs, 3 FTUCAs, and 5 others per- and polyfluoroalkyl substances)	Long chain per- and polyfluoroalkyl substances were detected higher than short chain. Frequently detected - PFCAs, FTCAs, and FTUCAs. Perfluorooctanoic acid - 32.5 ng/g. 6:2 fluorotelomer sulfonate (6:2 FTS) - 6 ng/g (67%). perfluorohexadecanoic acid (PFHxDA) - 107 ng/g.	[10]
LC-MS	Restek Raptor C18 column Particle size - 2.7 µm Temp - 40˚C Flow rate - 0.4 ml/min Injection volume - 5 µL MRM method in negative mode Run time - 10 min	23 per- and polyfluoroalkyl substances	23 per- and polyfluoroalkyl substances	Longer chains (C6-C14) were identified. Perfluorooctanoic acid - 0.04 ng/g (90%) Perfluorooctane sulfonic acid - 0.07 ng/g (62%) PFHxA - 0.02 ng/g (33%) Perfluoro butane sulfonate - 0.15 ng/g (29%) PFTrDA - 0.04ng/g (14%) PFTeDA and perfluoro decanoic acid - 0.06 and 0.02 ng/g (2 samples) PFUdA and 8:2 fluorotelomer sulfonate (8:2 FTS) - 0.01 and 0.03 ng/g (1 sample). When compared to other per- and polyfluoroalkyl substances, perfluoro butane sulfonate shows a higher concentration.	[7]
LC-HRMS	C18 column Particle size - 2.7 µm Temp - 40˚C Flow rate - 0.4 ml/min Injection volume - 20 µL Run time - 15 min	8 different compound classes	14 per- and polyfluoroalkyl substances (8:2/10:2 diPAP, 10:2/10:2 diPAP. 10:2/12:2 diPAP, 6:2/6:2 diPAP, 6:2/8:2 diPAP, 8:2/8:2 diPAP, 8:2 fluorotelomer sulfonates (8:2 FTSA), 10:2 FTSA, 10:2/10:2 FTMAP, 6:2/6:2 FTMAP, 6:2/8:2 FTMAP, 8:2/8:2 FTMAP and 8:2/10:2 FTMAP)	Major contaminants - PFCAs (C7, C8) and FTSAs (C6, C8)	[4]
HPLC-ESI-Q-TRAP	C18 column Flow rate - 0.2 ml/min Temp - 35 °C Injection volume - 5 µL Run time - 20 min MRM mode	23 per- and polyfluoroalkyl substances	6 per- and polyfluoroalkyl substances (perfluorooctanoic acid, perfluorobutanoic acid, PFPeA, PFHxA, PFHpA, and PFDoS)	Perfluorooctanoic acid - b/w 0.004 and 0.099 mg kg^-1^. Highly contaminated samples - baking papers. Mostly short carbon chains (4 to 6) were detected.	[17]
UHPLC-IMS-QTOF	C18 column Particle size - 1.7 µm Flow rate - 0.3 ml/min Temp - 35 °C Injection volume - 10 µL	Not mentioned	11 per- and polyfluoroalkyl substances	Compounds identified in ESI as deprotonated adducts. PFSAs vary from PFCAs in that they lack the (2M H)-ion in their low-energy spectrum.	[14]
LC-HRMS	C18 column with guard column Particle size - 1.7 µm Temp - 50 °C Flow rate - 0.3 ml/min Injection volume - 5 µL Run time - 15 min MRM mode	35 per- and polyfluoroalkyl substances	35 per- and polyfluoroalkyl substances	Perfluoro butane sulfonate and 6:2 diPAP detected above LOQ. 6:2 diPAP - mostly detected.	[13]
LC-MS	C18 column with 5mm guard column. Particle size - 2.7 µm	Not mentioned	13 per- and polyfluoroalkyl substances - perfluorobutanoic acid, PFODA, PFPeA, PFHxA, perfluorooctanoic acid, perfluorononanoic acid, perfluoro decanoic acid, PFUnDA, PFDoDA, PFTrDA, PFTeDA, PFHpA and PFHxDA. 4 per- and polyfluoroalkyl substances - perfluoro butane sulfonate, Perfluoro hexane sulfonate, perfluorooctane sulfonic acid, and PFDS	PFCAs - 22 to 56% detected. PFSs - 0 to 39% detected. The majority of detected PFCAs - perfluorobutanoic acid, perfluorooctanoic acid, perfluoro decanoic acid, and PFHxA with the carbon chains C8-C10	[1]

Hoang et al. utilized LC-HRMS to analyze extracts from hamburger wrappers, chips, cookies, and instant noodles. The separation process was carried out using a C18 column equipped with a guard column, featuring a particle size of 2.7 µm [1].

Stroski et al. employed an LC system connected to a Sciex QTRAP MS, utilizing a C18 column with a particle size of 1.7 µm. The gradient system consisted of solvent A (H_2_O: MeOH in a 95:5 ratio with ammonium acetate) and solvent B (MeOH with ammonium acetate), at a flow rate of 0.3 mL/min. The sample was injected at 5 µL, and the total experiment duration was 15 minutes [13].

Zabaleta et al. conducted the analysis using LC-QToF-MS. The LC system was connected to a 6530 QToF-MS with an ESI source, using an ACE UltraCore 2.5 Super C18 column at 35°C. The gradient system comprised Milli-Q water: MeOH with NH4Ac (eluent A) and MeOH/water (eluent B), both at a 95:5 ratio, with a flow rate of 0.3 mL/min and a 10 µL sample injection volume. A total of 46 PFAS were determined, with perfluorooctanoic acid being the most commonly identified compound across all samples. Only perfluorodecanoic acid was detected in the Indian samples, with a concentration of 14 ng/g [11].

Jovanović et al. employed LC connected to a triple quadrupole mass spectrometer for their analysis. The paper samples were extracted and analyzed using a Restek Raptor C18 column at 40°C, with a particle size of 2.7 µm. The gradient system included phase A (ammonium acetate) and phase B (MeOH) with a flow rate of 0.4 mL/min. A 5 µL sample was injected. Out of 23 PFAS identified, perfluorobutane sulfonate was found at higher concentrations compared to other PFAS. Compounds with carbon chain lengths of C6 to C14 were identified, with perfluorooctanoic acid being the most frequently detected [7].

Dueñas-Mas et al. performed the analysis using LC-HRMS on extracts from microwave popcorn bags, wrappers, and ice cream cups. They used a C18 XP column, specifically designed for high pH solvents, with a particle size of 2.5 µm, operating in negative mode. The mobile phase included ammonium acetate (solvent A) and MeOH (solvent B) with a flow rate of 0.3 mL/min. Short-chain PFCAs (C4 to C6) were identified in higher concentrations, although their detection frequency was lower. Longer-chain PFCAs were detected more frequently [3].

UHPLC-MS

Vera et al. performed the analysis using a UHPLC (I-Class) connected to an IMS QTOF MS with an ESI source. They employed a UHPLC BEH C18 column with a particle size of 1.7 µm at 35°C and a flow rate of 0.3 mL/min. The mobile phase consisted of phase A (water/acetonitrile, 90:10 v/v with ammonium acetate) and phase B (MeOH/acetonitrile, 60:40 v/v). A 10 µL sample was injected, and 11 PFAS were analyzed [14].

Chen et al. used UHPLC coupled to a triple quadrupole mass spectrometer with a C18 column operating at 40°C and a particle size of 1.8 µm. The separation was performed in reverse phase with a gradient system and analyzed in the mass range of m/z 50-1700 Da. The data was processed using MSConvert and R software. Of the 57 targeted PFAS, 26 were detected in popcorn bags (100%), paper bowls (72%), and other FPM (53%) [10].

Vavrouš et al. employed UHPLC connected to a triple quadrupole MS with an electrospray ionization source. The separation was carried out using a C18 column at 40°C with a mobile phase consisting of phase A (demineralized water with ammonium acetate) and phase B (chloroform/MeOH). The sample was injected at a volume of 3 µL [18].

Bugsel et al., Vavrouš et al., Surma et al., Zabaleta et al., Simonetti et al., Jovanović et al., Vera et al., Dueñas-Mas et al., Chen et al., Hoang et al., and Stroski et al. used similar analytical methods (LC-MS/UHPLC-MS) with different solvents. Notably, Simonetti et al., Jovanović et al., Dueñas-Mas et al., and Stroski et al. employed the same solvents (ammonium acetate and MeOH) for analysis.

**Figure 3:**
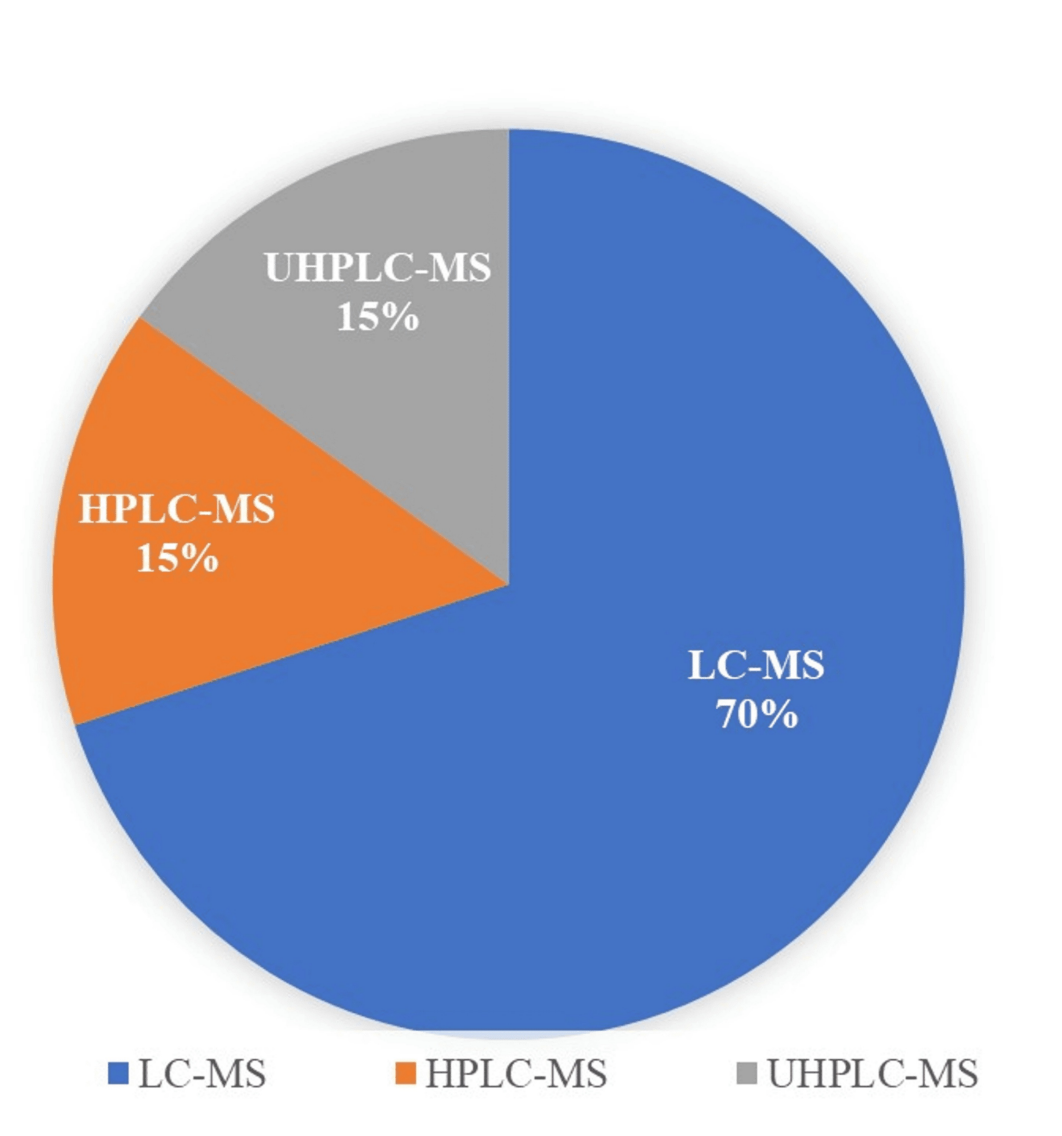
Analytical methods available for detection of per- and polyfluoroalkyl substances Image Credit: Original illustration. Created with MS Word LC-MS: liquid chromatography with mass spectrometer; HPLC-MS: high-performance liquid chromatography with mass spectrometer; UHPLC-MS: ultra-high performance liquid chromatography with mass spectrometer

Figure 3 depicts the overall analytical methods available for detecting PFAS.

Novel method for PFAS detection

An innovative method for determining PFAS in FPM is paper spray-mass spectrometry (PS-MS). This new approach allows for direct analysis of samples without modification, making it both time-efficient and sensitive. Unlike traditional methods that use electrospray ionization, PS-MS employs paper spray (PS), desalting PS (DPS), and nanoelectrospray ionization (nano ESI) for ionization. The system includes a C18 column and operates with a capillary inlet temperature of 250°C.

For analysis, samples such as microwave popcorn bags, Burger King French fry boxes, instant noodle boxes, McDonald's burger wrappers, and Burger King wrappers were cut into triangular pieces. The paper samples were placed in front of the MS inlet, where a 3.5 kV voltage was applied to facilitate ionization. MeOH was added directly to the paper to extract and elute the PFAS.

If the paper contains salts, a desalting procedure is required. This involves adding ultrapure water to the paper three times to remove salts and other contaminants. The desalting paper was then placed in front of the MS inlet for analysis. The samples were analyzed using UPLC-MS with solvent A (ammonium acetate) and solvent B (MeOH) as the mobile phases, operating at a flow rate of 0.3 mL/min for a total run time of 18 minutes.

The method successfully detected various PFAS compounds, including perfluoropentanoic acid (PFPeA), perfluorobutane sulfonate, PFHxA, 5:3 FTUCA, 5:3 FTCA, 6:2 FTCA, perfluoroheptanoic acid (PFHpA), perfluorononanoic acid, PFOS, perfluorooctanoic acid, and 6:2 FTUCA [19].

Case studies

Simonetti et al. utilized HPLC-ESI-Q-TRAP for analyzing PFAS in FPM, including microwave trays, plates, cups, and baking papers from fast food outlets and supermarkets. The samples underwent weighing, drying via rotary evaporator, and extraction with n-hexane/ethyl acetate and isopropanol/MeOH (90:10). Analysis was performed using a mobile phase of ammonium acetate and MeOH (45:55). Detection limits (LOD) ranged from 0.001 to 1.931 µg/kg, and quantification limits (LOQ) were between 0.004 and 5.792 µg/kg. The concentrations found were between 0.004 and 0.099 mg/kg. Among the 23 PFAS targeted, six were analyzed. Short-chain compounds (C4-C6) predominated, with perfluorooctanoic acid detected between 0.004 and 0.099 mg/kg. PFHxA was notably persistent, bioaccumulative, and toxic [17].

Chen et al. employed UHPLC-MS to detect PFAS in FPM like microwave popcorn bags, paper cups, and baking papers from supermarkets and online shops in Guangzhou, China. The paper samples were chopped, ultrasonicated, centrifuged, dried with nitrogen, reconstituted, filtered, and extracted by SPE. Average and median concentrations were 265 and 333 ng/g, respectively, with LODs ranging from 0.003 to 1.99 ng/g and LOQs from 0.01 to 6.62 ng/g. Of the 57 PFAS analyzed, 26 were detected in microwave popcorn bags (100%), paper bowls (72%), and other food contact materials (53%). PFAS levels were higher in products made with sugarcane pulp or other plant fibers compared to PE-coated paper. The most frequently identified compounds were PFCAs, FTCAs, and FTUCAs with longer carbon chains, and 6:2 FTS was detected at a rate of 67% [10].

Vera et al. used UHPLC-IMS-QTOF for PFAS analysis in cardboard, compact paper, and recycled cardboard from Spain and China. Two extraction methods were employed: first, ethanol extraction with ultrasonication followed by concentration, and second, a similar process with three ethanol extractions. Analysis was conducted with a mobile phase of water/acetonitrile (90:10 with ammonium acetate) and MeOH/acetonitrile (60:40) [14].

Langberg et al. performed PFAS analysis on paper products from Norway using UHPLC-QqQ-MS. Samples were extracted by ultrasound-assisted extraction (UAE) with ethyl acetate and analyzed. Out of 37 PFAS, 14 were detected with concentrations ranging from 0.5 to 971 µg/kg [12].

Jovanović et al. analyzed PFAS in FPM like microwave popcorn bags and pizza boxes using LC-MS. Samples were extracted with ASE using MeOH, evaporated, reconstituted, and filtered. LODs were between 0.025 and 0.1 ng/g, with PFAS concentrations ranging from 0.01 to 5 ng/mL. Longer-chain compounds (C6-C14) were identified. Perfluorooctanoic acid was detected at 0.04 ng/g (90% frequency), while PFOS was at 0.07 ng/g (62% frequency). PFHxA was identified at 0.02 ng/g (33%) and perfluoro butane sulfonate at 0.15 ng/g (29%) [7].

Hoang et al. used LC-MS to analyze PFAS in various FPM collected in Hanoi, Vietnam. Samples were ultrasonicated, centrifuged, concentrated, and analyzed. About 13 PFCA were identified with a method detection limit (MDL) of 0.040 to 0.10 ng/g. PFAS concentrations ranged from 372 to 624 ng/g in mochi paper trays [1].

Schwartz-Narbonne et al. conducted PFAS analysis on sandwich wrappers, popcorn bags, and other food packaging using UHPLC-ESI-HRMS. Selected samples were analyzed with 5-14 PFAS detected, with concentrations ranging from 55 to 7180 ng/g [5].

Stroski et al. sampled food packaging bags in Philadelphia, USA, using LCMS-HRMS. Samples were processed with MeOH, sonicated, centrifuged, and analyzed. Thirty-five PFAS were quantified, with concentrations from 0.5 to 26.6 ng/g. The most frequently detected compound was 6:2 diPAP, while perfluorobutane sulfonate was found in higher concentrations [13].

Bugsel et al. analyzed PFAS in impregnated paper samples from the Fraunhofer Institute for Process Engineering and Packaging using LC-HRMS. Samples were soaked in MeOH, evaporated, and analyzed with a mobile phase of water/MeOH (95:5) and MeOH [4].

In Poland, FPM were analyzed for PFAS using LC-MS. Samples were cut, soaked in acetonitrile, and analyzed with a mobile phase of water/formic acid and CAN/formic acid. PFAS concentrations ranged from 0.04 to 5 ng/ml, with LOQs between 0.02 and 0.17 pg/cm^2^. Ten PFAS, including perfluorooctanoic acid and PFOS, were detected, with PFOS and perfluorooctanoic acid being predominant [2].

Zabaleta et al. analyzed PFAS in microwave popcorn bags from various countries using LC-QToF-MS. The samples were extracted using focused ultrasonic solid-liquid extraction (FULSE), filtered, and analyzed. Method detection limits ranged from 4 to 27 ng/g. Forty-six PFAS were analyzed, with perfluoro decanoic acid detected at 14 ng/g in Indian samples, and short-chain PFCAs were prevalent in European samples [11].

Vavrouš et al. used HPLC-MS to analyze FPM in the Czech Republic. Two extraction methods were applied: ultrasonic extraction with acetonitrile and water, and LLE with inorganic salts. Extracted samples were centrifuged and analyzed with mobile phases of ammonium acetate in ultrapure water and MeOH. LOQ ranged from 0.0013 to 0.22 mg/kg with recovery rates of 70% to 120% [16].

In Egypt, PFAS in FPM were analyzed using LC-MS. Samples were extracted with pressurized liquid extraction and analyzed. LODs ranged from 3.9 to 30 pg, with perfluorooctanoic acid and PFOS detected in 79% and 58% of samples, respectively. Other PFAS identified included PFHpA, PFHxA, perfluorononanoic acid, and perfluorodecanoic acid [20].

FPM were analyzed for PFAS using HPLC-MS. Samples from the Czech Republic were extracted with LLE and analyzed with a mobile phase of demineralized water with ammonium acetate and MeOH. LOQs were 1.4 to 2.7 ng/g, with 41 PFAS detected [18].

Yuan et al. analyzed PFAS in FPM in China using LC-MS. Samples including baking paper and plastic bags were extracted using NMLEE. Eighteen PFAS were analyzed, with LODs from 0.002 to 0.03 µg/kg and LOQs from 0.01 to 0.2 µg/kg. Concentrations were around 10 ng/ml [15].

The overview of PFAS, including their extraction methods and analytical techniques, is summarized in Table 2.

**Table 2:** Overview of samples with their extraction methods and analytical techniques UHPLC-MS: ultra-high performance liquid chromatography with mass spectrometer; UHPLC-QqQ-MS: ultra-high performance liquid chromatography with triple quadrupole mass spectrometry; LC-MS: liquid chromatography with mass spectrometry; UHPLC-ESI-HRMS: ultra-high performance liquid chromatography with electron spray ionization high-resolution mass spectrometry; LC-HRMS: liquid chromatography with high-resolution mass spectrometry; HPLC-MS: high-performance liquid chromatography with mass spectrometry; LC-QTOF-MS: liquid chromatography-quadrupole time-of-flight tandem mass spectrometry

S. No.	Samples	Extraction	Analytical method and mobile phase	LOD/MDL	LOQ/MQL	Concentration	Reference
1	Microwave tray, plate, cup, baking papers A, B, C, and food box	Isopropanol/methanol [90:10]	HPLC-ESI-Q-TRAP, and ammonium acetate and MeOH (45:55)	0.001 to 1.931 µg kg^-1^	0.004 to 5.792 µg kg^-1^	0.004 and 0.099 mg kg^-1^	[17]
2	39 samples of microwave popcorn bags, baking paper, paper bowls, paper cups, and straws	Ultrasonication	UHPLC-MS	0.003 to 1.99 ng/g	0.01 to 6.62 ng/g	265 ng/g (average) and 333 ng/g (median)	[10]
3	Cardboard, compact paper, and recycled cardboard	Ultrasonication	UHPLC- IMS-QTOF and water/acetonitrile in the ratio 90:10 with ammonium acetate (phase A), methanol/acetonitrile in the ratio 60:40 (phase B)	-	-	-	[14]
4	10 paper products	Ultrasound-assisted extraction by ethyl acetate	UHPLC-QqQ-MS	-	-	0.5 and 971 µg kg^-1^	[12]
5	Microwave popcorn bags, fast food wrappers, pizza boxes and sandwich wrappers	Accelerated solvent extraction by methanol	LC-MS and ammonium acetate in water (phase A) and methanol (phase B)	-	between 0.025 and 0.1 ng g^-1^	between 0.01 and 5 ng mL^-1^	[7]
6	Instant noodle cup, chip wrapper, paper cup, hamburger wrapper, cookies wrapper and potato snack box	Ultrasonication	LC-MS	0.040 to 0.10 ng/g	-	-	[1]
7	Sandwich and burger wrappers, popcorn bags, dessert and bread wrappers	-	UHPLC-ESI-HRMS	-	-	55 to 7180 ng/g	[5]
8	18 samples of sandwich bags, snack bags, zipper seal bags, slider storage bags	Sonication	LC-HRMS and phase A - H_2_O: MeOH in the ratio 95:5 with ammonium acetate and phase B - MeOH with ammonium acetate	-	-	0.5 to 26.6 ng/g	[13]
9	Microwave popcorn bags, cardboard cup, ice cream cup, baking paper, burger wrapper, muffin cup, and takeaway paper bag	Sonication with MeOH	LC-MS and phase A - ammonium acetate and phase B - MeOH	0.0005 to 0.005 ng g^-1^	0.002 to 0.02 ng g^-1^	-	[3]
10	Baking paper, instant noodles cup, tinfoil, popcorn bag, plastic bag	Nonaqueous miscible liquid-liquid electroextraction (NMLEE)	LC-MS	0.002 to 0.03 μg·kg^-1^	0.01 to 0.2 μg·kg^-1^	10 ng·ml^-1^	[15]
11	Impregnated paper	MeOH	LC-HRMS and phase A - H_2_O/MeOH (95:5) with NH_4_Ac, phase B – MeOH/H_2_O (95:5) with NH_4_Ac	-	-	-	[4]
12	Wrapping papers	Liquid-liquid extraction (LLE)	HPLC-MS and of phase A - demineralized water with ammonium acetate, phase B - Chromasolv methanol	-	1.4 to 2.7 ng/g	-	[18]
13	Microwave popcorn bags	Focused ultrasonic solid-liquid extraction (FUSLE)	LC-QToF-MS and solvent A - Milli-Q water: MeOH in the ratio of 95:5 with NH_4_Ac and 1-MP and solvent B – MeOH: Milli-Q water with NH_4_Ac and 1-MP	4 to 27 ng/g	-	-	[11]
14	Papers	i) Ultrasonic extraction with acetonitrile and water; ii) liquid-liquid extraction with inorganic salts	HPLC-MS and solvent A - ammonium acetate in ultrapure water and solvent B - ammonium acetate in methanol	-	0.0013 to 0.22 mg/kg	-	[16]
15	17 samples of non-stick baking cups, Paper boxes, burger wrapping paper, microwave popcorn bags, and soup cups	Pressurized liquid extraction	LC-MS	3.9 to 30 pg	-	2.40 ng g^-1^	[20]
16	Wrapping papers, breakfast bags, roasting bags, and baking papers	Acetonitrile	LC-MS and phase A - water/formic acid (99/1) and phase B - acetonitrile/formic acid (99/1)	0.01 to 0.05 pg/cm^2^	0.02 to 0.17 pg/cm^2^	0.04 to 5 ng/ml	[2]

Difficulties in detecting PFAS

Detecting PFAS in samples presents several challenges, primarily due to background laboratory contamination. Often, insufficient investigation into fluorochemical contamination in the lab environment results in background particles appearing in analytical blanks. Additionally, impurities in existing standards, matrix effects, and issues during sample clean-up complicate the analysis. Gas chromatography-mass spectrometry (GC-MS) is generally avoided because samples must be derivatized beforehand, which can alter their original state [2].

Most analytical methods for polyfluoroalkyl amides (PFAA) face limitations such as a restricted range of detectable PFAAs, inadequate sensitivity, poor performance, and inaccurate quantification without internal standards or blank matrices. The confirmation of PFAS is hindered by the lack of available analytical standards. The results underscore the importance of using laboratory-grade materials to minimize potential contamination [13].

Discussion

The review examines various extraction and analytical methods for quantifying PFAS. Case studies highlight that perfluorooctanoic acid and PFOS are frequently detected. The extraction methods discussed include UAE, FUSLE, ASE, LLE, and NMLEE. UAE is widely used, but it requires internal standards to accurately analyze samples. In contrast, NMLEE can analyze samples without internal standards, making it a unique and preferable method for future analytical work. The solvents used for extraction include CAN, MeOH, ethyl acetate, ethanol, and MTBE.

The analytical techniques reviewed are LC-MS, UHPLC-MS, and HPLC-MS, with LC-MS being the most favored for reliably identifying PFAS. The solvents for these methods include water, ethanol, MeOH, acetonitrile, formic acid, milli-Q water, ammonium acetate, demineralized water, and Chromasolv MeOH. Among the case studies, four used ammonium acetate and MeOH as mobile phases. Some solvents pose toxicity risks to both operators and the environment. Future research should prioritize the use of green or eco-friendly solvents for analysis.

PFAS can accumulate in the tissues of wildlife over time. Through the food chain, it can affect the overall living organisms. they can affect growth, reproduction, and immune function, leading to declines in populations and biodiversity. PFAS can contaminate surface water, soil, and groundwater leading to long-term environmental degradation. They are highly mobile in water, making their contamination spread widely and persist for decades in ecosystems. PFAS exposure is linked to cardiovascular diseases, such as hypertension, and metabolic disorders, including increased cholesterol levels and thyroid dysfunction in humans. Chronic exposure to PFAS has been associated with liver damage, as reflected in altered liver enzyme levels. There is growing concern that PFAS exposure during pregnancy and early childhood may negatively affect brain development.

## Conclusions

This review emphasizes the importance of PFAS and their implications. Despite their widespread presence in daily life, PFAS are highly hazardous chemicals that are poorly understood. The case studies revealed that PFAS are found in junk FPM, posing potential risks to human health. Various extraction and analytical methods have been used to analyze and quantify these substances. LC-HRMS is the most commonly employed technique due to its ability to reduce background laboratory contamination. However, some PFAS precursors remain undetectable. Addressing this issue by reducing PFAS levels can enhance public health and prevent chronic diseases, thereby improving overall quality of life. Additionally, efforts should be made to minimize waste during extraction processes through advanced methodologies. Emerging techniques such as PS-MS, which enables direct sample analysis, may prove revolutionary and efficient methods for PFAS detection. Future research should develop advanced methods with high detection rates and shorter analysis times, improving analytical approaches for detecting PFAS precursors and using eco-friendly solvents to minimize environmental impact. Future research prospects focus on improving sensitivity and accuracy in detecting a wide range of PFAS compounds at ultra-trace levels in complex matrices. Developing databases and algorithms to improve the identification of unknown PFAS in environmental samples. Exploration of solvent-free or low-toxicity extraction methods. Developing time-series analysis techniques to track PFAS trends in various environmental matrices and modeling PFAS degradation pathways.
